# Difference in leaf water use efficiency/photosynthetic nitrogen use efficiency of Bt-cotton and its conventional peer

**DOI:** 10.1038/srep33539

**Published:** 2016-09-15

**Authors:** Ruqing Guo, Shucun Sun, Biao Liu

**Affiliations:** 1 Nanjing Institute of Environmental Sciences, Ministry of Environmental Protection, Nanjing 210042, China; 2 School of Life Sciences, Nanjing University, Nanjing 210093, China

## Abstract

This study is to test the effects of Bt gene introduction on the foliar water/nitrogen use efficiency in cotton. We measured leaf stomatal conductance, photosynthetic rate, and transpiration rate under light saturation condition at different stages of a conventional cultivar (zhongmian no. 16) and its counterpart Bt cultivar (zhongmian no. 30) that were cultured on three levels of fertilization, based on which leaf instantaneous water use efficiency was derived. Leaf nitrogen concentration was measured to calculate leaf photosynthetic nitrogen use efficiency, and leaf δ^13^C was used to characterize long term water use efficiency. Bt cultivar was found to have lower stomatal conductance, net photosynthetic rates and transpiration rates, but higher instantaneous and long time water use efficiency. In addition, foliar nitrogen concentration was found to be higher but net photosynthetic rate was lower in the mature leaves of Bt cultivar, which led to lower photosynthetic nitrogen use efficiency. This might result from the significant decrease of photosynthetic rate due to the decrease of stomatal conductance. In conclusion, our findings show that the introduction of Bt gene should significantly increase foliar water use efficiency but decrease leaf nitrogen use efficiency in cotton under no selective pressure.

China, as the largest cotton-producing country in the world with more than 2000 years’ history of cotton cultivation, holds approximately 25% of global annual production^1^. Yet, China loses 10% to 15% of its cotton production by insect pest each year. This proportion is even higher in serious years^2^. Therefore, Chinese scientists selected, bred and commercially cultivated Bt cotton since 1990s. The popularization and application of Bt cotton in China reduced the amount of chemical pesticide usage to 20~50% as compared to conventional cotton, alleviated environmental pollution caused by pesticide, protected beneficial insects in cotton field^2^.

However, Bt cotton brings some safety problems. First, transgenic Bt crops have been demonstrated to affect soil biota^3^,^4^,^5^,^6^,^7^ and gene flow between species or populations^8^,^9^,^10^,^11^, which is mostly adverse to functioning of natural ecosystems; second, although the control of Bt toxin over target insects and the positive effect on crop production^12^,^13^ have been well documented in previous studies^14^,^15^,^16^, the hidden dangers of selecting more resistant individuals from target insects^17^,^18^ or resulting in the break out of secondary insects and the decrease of crop production have also been proved^19^,^20^; third, the fabricated products of Bt cotton may have adverse effect to the health of human^2^.

Apart from the effects on external environments, the physiology of cotton has been proven affected by many studies^21^,^22^,^23^. However, studies about the Bt gene’s effect on resource allocation pattern from the life history perspective are scarce. From a basic assumption of life history theory, the increased allocation of resources to one function precludes the allocation of these functions to other functions^24^, we speculate that the introduced Bt gene may affect cotton’s water use and nitrogen use efficiencies. First, the transcription and translation of Bt gene consume resources that could have been used for plant growth and reproduction. Second, the expression of Bt protein may change the interaction between crop plants and microbe including ectomycorrhizal and endomycorrhizal fungi which may further change plants’ water/nutrient absorptive ability^25^,^26^,^27^. Third, Bt gene may have uncertain effects on the expression of the genes related to plant water and nutrient use efficiency by changing their expressive intensity, quantity or even enzyme activity^28^,^29^,^30^.

As the most important commercial crop in China, cotton is the main consumer of agricultural water^2^. The main cotton growing districts in China locate in the North China plain, Loess Plateau and middle reaches of the Yangtze River, majority of which suffers from drought in most time of a year^1^. China is already in moderate status of water shortage^31^, and among national annual water consumption, agricultural water use accounts for nearly 62%. Moreover, available water for agriculture is still in severe shortage. Out of nationwide 121.72 million hm^2^ arable land, only 58.47 million hm^2^ could be effectively irrigated^31^,^32^. Considering the large area of cotton plantation and low water use efficiency as little as 30% to 40%^2^, if Bt cotton truly has a relatively lower water use efficiency, the serious situation of water deficiency will undoubtedly worsen.

Not only the water supply problem, but the water quality problem in China is unprecedentedly dangerous as well. The situation is primarily owing to the eutrophification of waterbodies, especially within main crop-growing area such as Changjiang and Zhujiang Deltas^33^. Apart from the main consumer of agricultural water, cotton is also the main consumer of chemical fertilizer and the main source of agricultural non-point source pollution in China. If the nutrient use efficiency declines due to the introduction of Bt gene, more chemical fertilizer may have to be used to maintain cotton yield, which will intensify environmental pollutions around farmlands. Therefore, it is imperative to evaluate the Bt-gene introduction effects on water and nutrient use efficiency.

Leaves are the organ completing the processes of both water transpiration and CO_2_ sequestration; the ratio of photosynthetic rate to transpiration rate is often used to characterize instantaneous water use efficiency (IWUE). Similarly, leaf photosynthetic nitrogen use efficiency (PNUE) can be characterized as the ratio of photosynthetic rate to leaf nutrient concentration. Because the instantaneous water efficiency is not a life-time characteristic, long term water use efficiency (LWUE) should be examined. Carbon isotope composition (δ^13^C) has been widely used to characterize the LWUE because it is associated with the ratio of CO_2_ partial pressure within the leaves (Pi) to the CO_2_ partial pressure outside the leaves (P_amb_). Previous studies have extensively demonstrated a positive correlation between the long term efficiency and δ^13^C^34^,^35^.

In this study, leaf photosynthetic rate, transpiration rate, foliar total nitrogen concentration and δ^13^C were measured to calculate IWUE and PNUE and characterize LWUE. The objective of this study was to test whether the introduction of Bt-gene affects water use efficiency and nitrogen use efficiency in cotton.

## Materials and Methods

### Cotton plantation

Experiments with cotton plantation and field measurements were conducted in Ferry Village, Yudai Town, Luhe District, Nanjing (118°54′ E, 32°14′ N), eastern China in 2009. The study site is within northern subtropical monsoon climate zone. Mean annual temperature is 16.1 °C, with the monthly means in January and July being 1.5 °C and 29.0 °C, respectively. Mean annual precipitation is 975 mm, more than 70% of which occurs in summer between June and August.

The experiment was started at the middle of April and was ended in late October. Zhongmian no. 30 (Z30 for abbreviation), the genetically modified cotton expressing the Bacillus thuringiensis Cry1AA toxin and its compared isogenic conventional cultivar zhongmian no. 16 (Z16 for abbrevation) were used. In middle April, each seed was placed on the soil surface of an individual pot. The seedlings were transplanted to the farmland at the density of 30,000 individuals per hectare when majority of them reached approximately 15 cm in height. The farmland’s basal total nitrogen concentration was 2.35 g kg^−1^. For each cultivar, three fertilizer levels were set up as 50%, 100% and 150% of regular level according local peasant’s experience. Regular level was: base manure contained 15 tons of manure and 225 kg urea per hectare, additional manure contained 10 tons of manure and 150 kg urea per hectare. Thus, the factorial experiment consists of two factors (cultivar and fertilizer level) that had two and three levels, respectively, forming six treatments in total.

After transplantation, all managements including irrigation, pesticide and herbicide application were following the techniques of local peasants.

#### Physiological Measurements

Li-cor 6400XT portable photosynthesis system (Li-Cor, Lincoln, NE, USA) was used to measure and record photosynthetic parameters under saturated light conditions (photosynthetic active radiation (PAR) was set as 1250 μmol·m^−2^·s^−1^) at three stages (flowering peak stage (FPS), the beginning of boll opening stage (BBOS), boll opening peak stage (BOPS)) of the growing season. During measurements, flow rate was set as constant 500 μmol s^−1^ and leaves were maintained at 33 °C, which was most frequently measured during the growing season of the study area. At FPS, 3 individuals from each treatment were randomly selected and one flag leaf of each individual was used to measure photosynthetic parameters including net photosynthetic rate (Pn, μmolCO_2_·m^−2^·s^−1^), transpiration rate (E, μmolH_2_O·m^−2^·s^−1^) and stomatal conductance (Gs, molH_2_O·m^−2^·s^−1^). Instantaneous water use efficiency (IWUE, μmolCO_2_/μmolH_2_O) is calculated as by dividing net photosynthetic rate by transpiration rate. 18 individuals across 6 treatments were randomly measured in sequence to avoid systematic error. All sampled individuals were tagged for the follow-up measurements. At BBOS and BOPS, we randomly selected one flag leave on each tagged individual to perform measurements. That is to say, for each tagged individual, different leaves were measured at different stages since it is hardly possible to keep all leaves alive during the whole growing season.

After the final measurement of the whole growing season, the measured leaves were sampled to measure foliar nitrogen content with H_2_SO_4_-H_2_O_2_-indophenol blue method. Photosynthetic nitrogen use efficiency (PNUE) was calculated as the ratio of Pn_max_ to foliar N content.

At each stage, soil of depth from 0–20 cm was sampled to measure soil water content and we found soil water content maintained stable across all treatments.

### Stable carbon isotope

For each treatment, 2 canopy leaves from each of three measured individuals (N = 6 for each treatment) were collected immediately after harvesting in mid-October, then dried to constant mass at 70 °C and ground in the mill to pass 0.147-mm mesh. The carbon isotope composition of leaves was analyzed at the stable isotope lab of Chinese Academy of Sciences (Beijing, China) using an isotope ratio mass spectrometer (DELTA ^plus^ XP, analytical precision was about 0.1‰). The results were then expressed as δ^13^C values to characterize leaf long-term water use efficiency (LWUE), using a formula as^34^:

δ^13^C = (R_sam_ − R_std_)/R_std_ × 1000‰, where sam and std refer to sample and standard material, respectively.

### Statistical analysis

To evaluate the effects of cultivar, fertilizer level, and development stage on IWUE, PNUE, and δ^13^C values, as well as some other traits, three or two-way ANOVAs (for some traits without the effect of the stage) were conducted, and post-hoc Fisher-LSD tests were used to determine the significance level of the difference between different treatments. Homogeneity of variance was checked at the first step to ensure the validity of ANOVA tests. All the statistical analyses were run by JMP10.0 software packages.

## Results

### Instantaneous water use efficiency

Three-way ANOVAs revealed significant effects of cultivar, development stage and fertilizer level on Pn_max_, E, IWUE and Gs (For Pn_max_: cultivar: F_1,36_ = 112.50, p < 0.001; stage: F_2,36_ = 56.07, p < 0.001; fertilizer level: F_2,36_ = 214.10, p < 0.001; For E: cultivar: F_1,36_ = 331.69, p < 0.001; stage: F_2,36_ = 115.22, p < 0.001; fertilizer level: F_2,36_ = 12.72, p < 0.001; For IWUE: cultivar: F_1,36_ = 169.29, p < 0.001; stage: F_2,36_ = 88.49, p < 0.001; fertilizer level: F_2,36_ = 85.07, p < 0.001; For Gs: cultivar: F_1,36_ = 248.78, p < 0.001; stage: F_2,36_ = 35.67, p < 0.001; fertilizer level: F_2,36_ = 33.25, p < 0.001). Z16 generally had higher Pn_max_ than Z30, especially at the flowering peak stage. For both cultivars, Pn_max_ were higher at the beginning of boll opening stage than those at the flowering stage and boll opening peak stage. Moreover, higher fertilizer level enhanced Pn_max_ for both Z16 and Z30 (Table 1).

Z16 had higher E than Z30 at all three stages. Generally, E remained relatively stable at flowering peak stage and boll opening peak stage but was higher at the beginning of boll opening stage, especially for Z30. For Z16, fertilizer level did not have substantial effect on E, but at the level of low fertilizer supply, E was also lower at flowering peak stage and boll opening peak stage for Z30 (Table 1).

Similar as E, Z16 had higher Gs and Z30 showed apparent low Gs at flowering peak stage and boll opening peak stage when fertilizer level was low. Pearson correlation was performed and detected strong correlation between Pn and Gs (r = 0.778). Correlation between E and Gs was even stronger (r = 0.847).

For IWUE, cultivar, fertilizer level, stage and all interactions had significant effects. Generally, Z30 had significantly higher IWUE than Z16. IWUE was significantly enhanced by higher fertilizer level. Interestingly, Z30 at the level of low fertilizer supply showed extremely high IWUE at boll opening peak stage (Table 1).

### Carbon isotope composition (δ^13^C)

**Figure 1:**
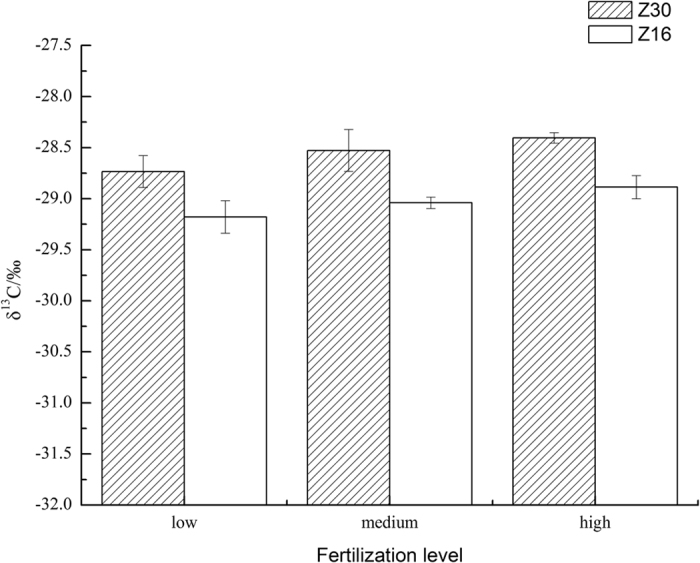
Foliar δ^13^C of Z30 and Z16 under three fertilizer levels. Z30: shaded columns; Z16: blank columns. The errors bars denote 1 standard error. N = 6 for each treatment.

Z30 had significantly higher *δ*^*13*^*C* than Z16 but neither fertilizer level nor interaction between cultivar and fertilizer level had significant effect on *δ*^*13*^*C* (cultivar: F_1,30_ = 18.8, p = 0.001; fertilizer level: F_2,30_ = 2.6, p = 0.087; cultivar*fertilizer level: F_2,30_ = 0.04, p = 0.969; Fig. 1). Fisher-LSD test showed Z30 had obviously higher δ^13^C than Z16 at all three stages. Higher fertilizer levels seemed could enhance δ^13^C slightly but the effect was insignificant.

### Photosynthetic nitrogen use efficiency

**Figure 2:**
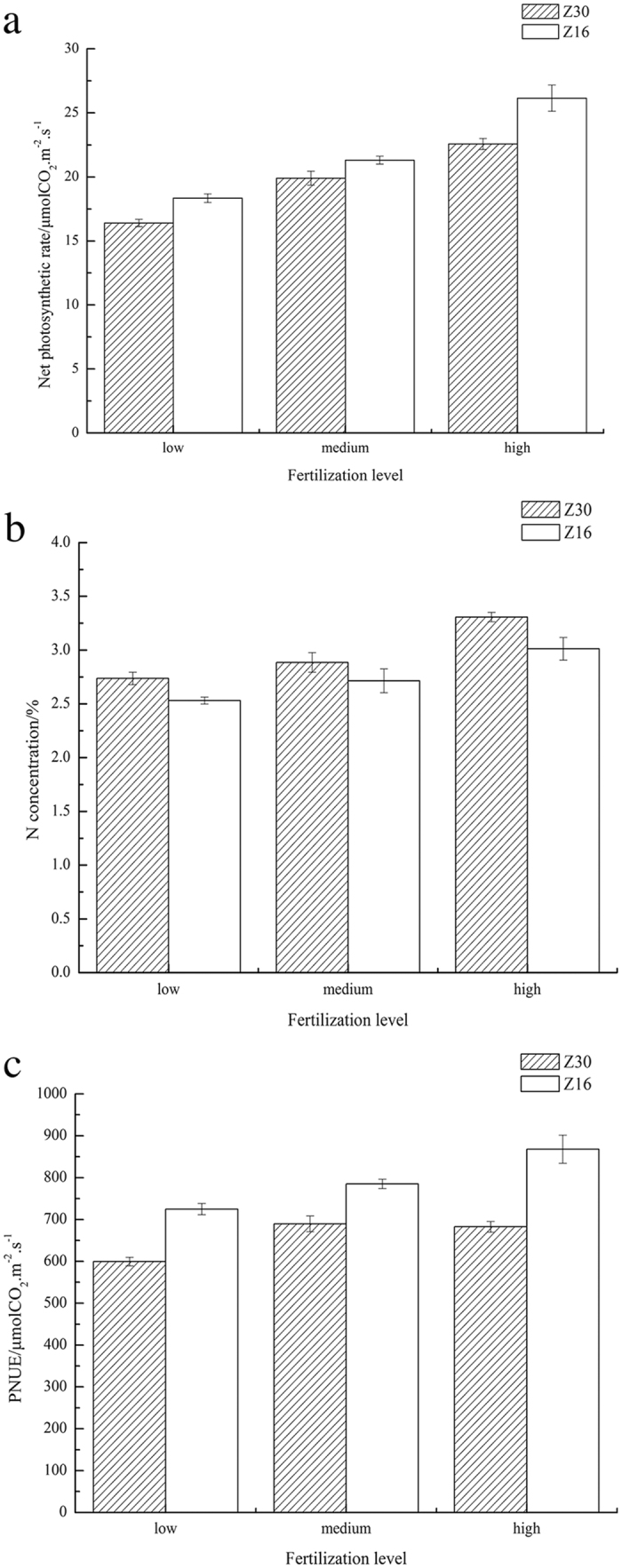
Net light-saturated photosynthetic rates. (**a**) Foliar total N concentrations (**b**) and photosynthetic nitrogen use efficiencies (**c**) of Z30 and Z16 under three fertilizer levels at boll opening peak stage. Z30: shaded columns; Z16: blank columns. The errors bars denote 1 standard error. N = 3 for each treatment.

At the boll opening peak stage, the only stage at which PNUE was examined, the introduction of Bt gene decreased Pn_max_ but enhanced foliar nitrogen content (Fig. 2a,b). Therefore, Bt gene decreased cotton’s PNUE as the ratio of Pn_max_ to foliar nitrogen concentration. For both cultivars, PNUE was significantly lower at low fertilizer level than at medium and high level, whereas no difference was detected between the latter two stages. Meanwhile, at all three fertilizer levels, Z30 had lower PNUE compared to Z16 (F_1,12_ = 79.506, p < 0.001), suggesting the interaction between cultivar and fertilizer level was insignificant (Fig. 2c).

## Discussions

### Leaf water use efficiency

IWUE, as defined above, is determined by both Pn_max_ and E. We may find the source of difference in IWUE by inspecting the difference in Pn_max_ and E between Z30 and Z16. Generally, the introduction of Bt gene decreased both Pn_max_ and E. At the flowering peak stage, Z30’s Pn_max_ was 39% of Z16’s at the level of low fertilization. At the level of medium and high fertilization, this number was 76% and 71%. Meanwhile, Z30’s E was 30% of Z16’s at the level of low fertilization. At the level of medium and high fertilization, this number was 66% and 59%. In another word, compared to Pn, Z30’s E reduced with more extent. The situation was similar for both beginning of boll opening stage and boll opening peak stage. Both Pn_max_ and E showed a strong positive correlation with Gs, which was consistent with previous studies^36^,^37^. Compared to Z16, Z30’s lower Gs was consistent with the inter-cultivar difference of Pn_max_ and E. Furthermore, relative to H_2_O, CO_2_ in the leaves has an additional diffusive resistance^38^, suggesting that the decrease of Gs would lead to less decrease in Pn_max_ than in E^36^,^37^. Rosenthal *et al*. also made the same conclusion in their study about cotton and sorghum^39^. They found the reduction of Gs would decrease both E and Pn but the reduction of Pn was not as obvious as E’s reduction. Although Rosenthal’s work mainly focused on the water deficit’s effect while our work focused on the effect of Bt gene’s introduction, we found similar changes of photosynthetic parameters. Therefore, we suggest Z30’s advantage on IWUE was after all resulted from the decrease Gs which led to the asynchronous reductions of Pn and E. The same pattern was found in another study of ours in Bt rice^40^. However, we do not know whether the decrease of Gs was related to an obvious change of foliar structure and a comparative dissection of both Z30 and Z16 leaves is helpful to uncover the mechanism of decreased Gs.

Foliar δ^13^C is a common and useful index of seasonally integrated, namely long-term WUE. Generally, δ^13^C is positively correlated to LWUE^34^,^35^. In our study, significantly higher δ^13^C value, namely higher LWUE in Z30 was consistent with the result of IWUE. This suggested the introduction of Bt gene truly enhanced water use efficiency of cotton. A previous study found differences in soil water content between Bt- and non-Bt-maize^41^, but we did not detect this difference after running a 3-way anova to explore if any factor might affect soil water content (cultivar: F_1,48_ = 0.9337, p = 0.3387; fertilizer level: F_2,48_ = 0.2421, p = 0.7859; stage: F_2,48_ = 1.6945, p = 0.1945). Therefore, we suggest the measured differences of photosynthetic parameters were not due to the deviation of soil water content but a direct consequence of the introduction of Bt gene.

With the advantage of both IWUE and LWUE, Z30 may be suitable for plantation in arid areas. This is important to use arid farmlands to enlarge planting areas and promote economic development. However, the advantage of higher IWUE is companying with the cost of decreased Pn and in theory would negatively affect growth and production. Indeed, we measured above-ground biomass and found Z30’s biomass was smaller than Z16’s although the difference was not significant (data not shown). Therefore, in terms of the overall situation, we suggest the introduction of Bt gene brings advantages that outweigh disadvantages.

### Photosynthetic nitrogen use efficiency

As IWUE, PNUE is a ratio determined simultaneously by numerator (Pn_max_) and denominator (foliar nitrogen concentration). Within cultivars, both Pn_max_ and N concentration increased with more fertilizer supply but the amplitude of Pn_max_ was bigger than foliar N. This result means PNUE positively correlated with N concentration within both Bt and conventional cultivars. The pattern is consistent with a previous study^42^ but is incompatible with another^43^.

Z16, which had higher Pn_max_ but lower N concentration, showed higher PNUE than Z30. This is contrast to the well-known tendency that Pn_max_ is generally positively related to mass-based foliar N concentration^44^,^45^,^46^,^47^. We suppose this unusual result is partly because of the relatively less photosynthetic nitrogen (here we mean the nitrogen participated in photosynthesis in the form of protein and enzymes) in Bt cotton, although it had higher foliar total nitrogen concentration relative to the conventional cultivar. Photosynthesis is an enzyme-mediated process that largely depends on Ribulose-1, 5-bisphosphate carboxylase-oxygenase (Rubisco enzyme). Rubisco enzyme, as the key enzyme that accounts for up to 30% of total leaf nitrogen, is often positively correlated to nitrogen availability and could directly influence photosynthetic capacity^44^,^48^,^49^. Bt cotton, which allocates part of its nitrogen resource to construct Bt protein that did not exist in conventional cotton, might have less available nitrogen to construct Rubisco enzyme and other nitrogen-demanded materials involved in photosynthesis. This explanation is similar to the result of Field & Mooney^44^ who claimed the expression of N-containing secondary compounds for defense against predation would lead to the decline of PNUE. However, nitrogen competition should not be the main factor affects PNUE in this study. According to a previous study involving a comparison of total protein content across 107 plants, protein in cotton leaves was about 1.26% of total weight^50^, which means 0.38% (3800 ppm) of total foliar weight was Rubisco enzyme. Assuming Bt protein content in cotton leaves is similar to that in maize^51^, namely 42 ppm, then the decrease of Rubisco enzyme due to the nitrogen competition by Bt cotton is just about 1%. Therefore, the obvious decrease of Pn in Z30 cannot be fully explained by the production of Bt protein. We infer the decrease of Gs might be the main factor lowered Pn since CO_2_ supply would be limited under this circumstance. In another study^40^, we found Gs of Bt rice decreased compared to conventional rice. Pu^52^ also found Bt cotton’s Gs decreased under both low and high selective pressures. Besides, Pu found chlorophyll content in Bt cotton was lower than that in conventional cotton. We did not measure chlorophyll content in our study, but considering Pu used the same cotton cultivar as we used, the decrease of chlorophyll content might also be a non-negligible factor led to the decrease of Pn in Bt cotton.

In our study, the enhancement of water use efficiency in Bt cotton was achieved by decreasing Gs. The reduction of Gs would limit CO_2_ supply in leaves and hence Pn_max_ also decreased, which further lead to the decrease of PNUE. That is to say, maintaining WUE may incur a cost of PNUE, as shown in several previous studies^53^,^54^,^55^. Because of the trade-off between WUE and PNUE, the advantage on WUE in Bt cotton could be neutralized by the disadvantage on PNUE, especially in those water-rich areas where water supply is not the major limiting factor for cotton plantation. But in arid areas where water is a limiting resource, Bt cotton would be advantageous and the decreased PNUE might be compensated by more fertilizer application.

To summarize, our results show that cotton displayed a significant lowering of Gs after introduction of the Bt gene. The decreased Gs enhanced WUE for the transgenic cultivar due to the asynchronous decrease of Pn_max_ and E. However, PNUE was decreased in the Bt cultivar due to the combinative effects of nitrogen trade-off and decrease of Gs and chlorophyll content. That is to say, with the advantage of saving water, it is possible that more fertilizer will be applied to Bt cotton to maintain normal yield and incur more severe agricultural non-point source pollution. Besides, considering the decreased Gs detected in this study and another study about Bt rice^40^ and observations from others of early aging and metabolic unbalance^2^, the risk of foliar structure damage cannot be neglected and need to arouse more attention.

## Additional Information

**How to cite this article**: Guo, R. *et al*. Difference in leaf water use efficiency/photosynthetic nitrogen use efficiency of Bt-cotton and its conventional peer. *Sci. Rep.* **6**, 33539; doi: 10.1038/srep33539 (2016).

## Figures and Tables

**Table 1:** Leaf instantaneous water use efficiency (IWUE μmolCO_2_/μmolH_2_O), net light-saturated photosynthetic rate (Pn_max,_ μmolCO_2_·m^−2^·s^−1^), transpiration rate (E, μmolH_2_O·m^−2^·s^−1^) and stomatal conductance (Gs, molH_2_O·m^−2^·s^−1^) of Z30 and Z16 under three fertilizer levels at different stages.

		Z30-Low	Z30-Medium	Z30-High	Z16-Low	Z16-Medium	Z16-High
FPS	IWUE	4.02 ± 0.20	4.45 ± 0.26	5.75 ± 0.17	3.04 ± 0.10	3.81 ± 0.12	4.83 ± 0.08
	Pn_max_	7.21 ± 0.29	15.42 ± 0.43	19.29 ± 1.08	18.34 ± 0.21	20.24 ± 0.41	27.36 ± 0.98
	E	1.80 ± 0.09	3.49 ± 0.23	3.36 ± 0.22	6.05 ± 0.22	5.31 ± 0.06	5.66 ± 0.17
	Gs	0.08 ± 0.00	0.19 ± 0.01	0.22 ± 0.01	0.45 ± 0.02	0.41 ± 0.02	0.38 ± 0.02
BBOS	IWUE	3.71 ± 0.07	3.87 ± 0.05	5.43 ± 0.16	2.87 ± 0.04	3.78 ± 0.06	4.70 ± 0.10
	Pn_max_	18.93 ± 0.31	21.88 ± 1.08	24.41 ± 0.84	18.35 ± 0.37	21.11 ± 0.57	28.55 ± 0.84
	E	5.11 ± 0.18	5.65 ± 0.24	4.51 ± 0.25	6.40 ± 0.16	5.59 ± 0.22	6.08 ± 0.31
	Gs	0.27 ± 0.01	0.35 ± 0.00	0.37 ± 0.01	0.36 ± 0.02	0.38 ± 0.01	0.53 ± 0.05
BOPS	IWUE	7.55 ± 0.39	4.65 ± 0.12	5.36 ± 0.10	3.97 ± 0.11	4.53 ± 0.07	4.94 ± 0.02
	Pn_max_	14.88 ± 0.81	19.63 ± 1.02	22.57 ± 0.43	17.81 ± 0.20	20.62 ± 0.58	23.10 ± 0.81
	E	1.97 ± 0.08	4.22 ± 0.18	4.21 ± 0.13	4.49 ± 0.16	4.55 ± 0.12	4.68 ± 0.17
	Gs	0.15 ± 0.01	0.32 ± 0.02	0.33 ± 0.00	0.37 ± 0.02	0.31 ± 0.04	0.39 ± 0.00
